# Supplementary material for: A Global Screen for Assembly State Changes of the Mitotic Proteome by SEC-SWATH-MS
Source: Cell Syst. 2020 Feb 26;10(2):133–155.e6. doi: 10.1016/j.cels.2020.01.001 (PMC7042714; doi:10.1016/j.cels.2020.01.001)

Q86VP6 | CAND1_HUMAN | CAND1 KIAA0829 TIP120 TIP120A
Monomer MW [kDa]: 136.376 Monomer expected elution fraction: 37

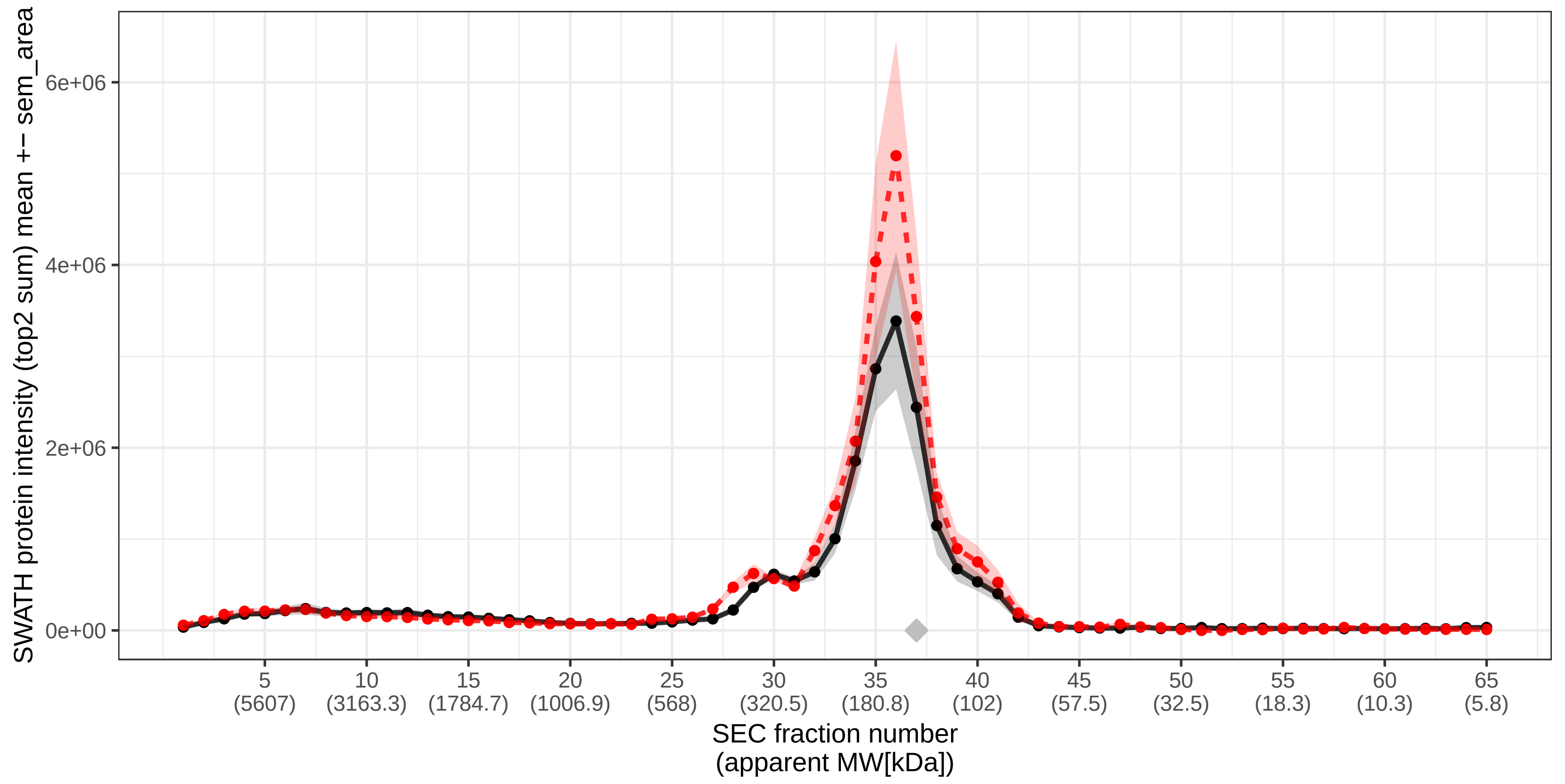

Supplement: Data S1. SEC-SWATH-MS Protein Chromatograms, Related to Figure 1 [file mmc6.zip › SECchrom_Q86VP6_CAND1_HUMAN_CAND1_KIAA0829_TIP120_TIP120A.pdf]
